# Assessment of C-reactive protein levels as an indicator for lung infiltrates in patients with COVID-19 pneumonia

**DOI:** 10.25122/jml-2023-0104

**Published:** 2023-07

**Authors:** Moaz Atef, Hoda Eid, Mohammad Amin, Mohamad Shehata, Ahmed Shaarawy, Osama Nabawy, Ahmed Wahba, Ahmed Aboseif, Mohamed Rakha, Mohamed Zaki, Eid Mohammed, Abdullah Albalsha, Sameh Nour, Amr Rezk, Mohamed Shaheen, Ahmed Kabil

**Affiliations:** 1 Department of Chest Diseases, Faculty of Medicine, Al-Azhar University, Cairo, Egypt.; 2 Department of Radiology, Faculty of Medicine, Al-Azhar University, Cairo, Egypt.; 3 Department of Clinical Pathology, Faculty of Medicine, Al-Azhar University, Cairo, Egypt

**Keywords:** COVID-19, lung infiltrates, CRP

## Abstract

Lung infiltrates are frequently observed in patients with COVID-19 infection and require specialized management. Identifying reliable laboratory parameters to reduce the need for chest CT scans in non-desaturation patients is of great interest. This study aimed to investigate the potential of C-reactive protein (CRP) as an indicator to identify the presence of lung infiltrates in early COVID-19 infection. The study was conducted at Al-Azhar University hospitals from May 2021 to March 2022 and included 210 patients with COVID-19 infection confirmed by positive PCR, all of whom were previously healthy, non-smokers, and non-hypoxemic. CRP levels were assessed and correlated with lung infiltrates observed in CT chest examinations. The mean value of CRP was 40.3±14.3 mg/L in males and 36.6±15.2 mg/L among females. One hundred sixty-two patients had pneumonic infiltrates, while 48 had no infiltrates. The mean value of CRP was 45.02±10.2 mg/L in patients with radiological infiltrates and 18.8±7.8 mg/L in patients without radiological infiltrates. Based on our findings, a CRP value greater than 29.8 mg/L was suggested as a cut-off value to indicate the presence of lung infiltrates. CRP is a simple laboratory marker that, at certain limits, may point to the presence of pneumonic infiltrates in early non-hypoxemic patients with COVID-19 infection.

## INTRODUCTION

C-reactive protein (CRP) is a well-known acute-phase reactant synthesized by various cells, including lymphocytes, adipocytes, and macrophages. However, it is primarily produced by hepatocytes [1]. When triggered by a range of non-specific inflammatory stimuli, CRP synthesis occurs rapidly, often mediated by the pro-inflammatory cytokine IL-6 [2]. CRP binds to the outer surface of the target stimuli through opsonization, leading to the subsequent activation of the complement system via the classic pathway, ultimately resulting in phagocytosis of the offending agent by the macrophages [3]. In the context of the COVID-19 pandemic, CRP was found to be positively correlated with disease severity and even suggested as a sensitive predictor of disease outcome and prognosis [4]. Despite its significance, there remains a lack of sufficient data to establish specific cut-off values for CRP. Therefore, this study aimed to address this gap by investigating whether CRP levels could be clearly associated with a specific cut-off value indicative of lung infiltrates in patients with early COVID-19 infection.

## MATERIAL AND METHODS

This study was conducted between May 2021 and March 2022 in the Chest Department of Bab-El-Shaaria, Al Zahraa, and Al-Hussein hospitals. A random sample of 210 patients with COVID-19 infection confirmed by positive PCR was included in the study. Patients who were smokers or had pulmonary and/or extra-pulmonary comorbidities were excluded to eliminate the influence of different conditions and diseases on CRP levels. All patients exhibited an abnormally elevated CRP (>10 mg/L).

CRP levels and CT chest scans were assessed between the fourth and sixth days from the onset of symptoms. All participants provided written informed consent before enrollment and underwent a comprehensive evaluation, including clinical history and examination, complete blood count (CBC), CRP levels, random blood sugar (RBS), liver and kidney function tests, PCR for COVID-19, and CT chest scans. Inclusion criteria included patients with positive PCR for COVID-19 and lower respiratory tract symptoms. Exclusion criteria included hypoxemic patients or those with chronic lung diseases such as interstitial lung disease (ILD). Patients with known comorbidities, such as hypertension, diabetes, cardiac, renal, or hepatic conditions, were also excluded.

## STATISTICAL ANALYSIS

Data were analyzed using the Statistical Package for the Social Sciences (SPSS) version 24. Quantitative data were expressed as mean±SD, representing the central value and dispersion of a discrete set of numbers, respectively. The mean is calculated as the sum of all values divided by the number of values in the set. Standard deviation (SD) measures the dispersion of values around the mean. A low SD indicates that the values are close to the mean, while a high SD suggests that the values are more widely spread.

For qualitative data, frequency and percentage were used for expression. The statistical methods were verified, assuming a significance level of p<0.05, which means that results with a p-value less than 0.05 were considered statistically significant. To compare between two means for abnormally distributed data, the Mann-Whitney U test (MW) was employed. The Receiver Operating Characteristic (ROC) curve was used to detect the cutoff value, sensitivity, specificity, positive predictive value (PPV), and negative predictive value (NPV).

## RESULT

Table 1 shows the descriptive data of all participants. Among the participants, 136 were males (64.8%) and 74 (35.2%) females. The mean age of patients was 39.7±13.04 years, with a minimum age of 18 years and a maximum age of 60 years. Regarding CT chest findings, 48 patients (22.9%) had normal scans, while 162 (77.1%) showed infiltrations.

**Table 1:** Descriptive data of participants

		Participants (N = 210)	
Sex	Male	136	64.8%
	Female	74	35.2%
Age (years)	Mean ±SD	39.7 ± 13.04	
	Min - Max	18 – 60	
CT	Normal	48	22.9%
	Infiltration	162	77.1%
CRP (mg/L)	Mean ±SD	39.04 ± 14.6	
	Min - Max	10.5 – 68	

The mean CRP of all participants was 39.04±14.6 mg/L with a minimum CRP level of 10.5 mg/L and a maximum CRP of 68 mg/L. There was no statistically significant difference in CRP values (p-value=0.055) between male and female patients (Table 2). The mean CRP level in male patients was 40.3±14.3 mg/L (median=44 mg/L), while it was 36.6±15.2 mg/L (median=38 mg/L) in female patients.

**Table 2:** Correlation between mean CRP value and sex of patients

		Sex		Stat. test	p-value
		Male (N=136)	Female (N=74)		
CRP (mg/L)	Mean ±SD	40.3±14.3	36.6±15.2	MW=4226	0.055 NS
	Median	44	38		

There was a highly statistically significant increase in CRP levels in patients with infiltration (45.02±10.2, median=48 mg/L) when compared with CRP levels in normal CT patients (18.8±7.8, median=17 mg/L) (Table 3).

**Table 3:** Correlation between CRP level and CT of patients

		CT		Stat. test	p-value
		Normal (N=48)	Infiltration (N=162)		
CRP (mg/L)	Mean ±SD	18.8±7.8	45.02±10.2	MW=245.5	< 0.001 HS
	Median	17	48		

**Figure 1:**
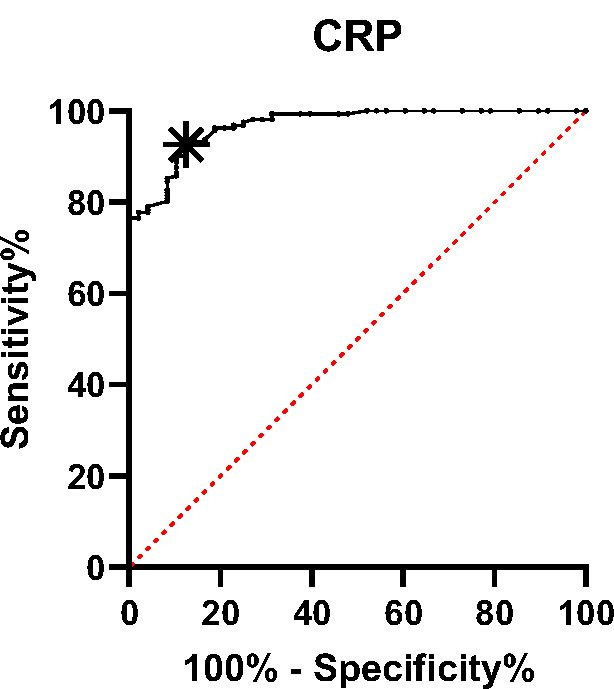
ROC curve for serum CRP in patients with and without CT infiltration

Using the ROC curve (Figure 1), serum CRP was shown to effectively discriminate between patients with and without CT infiltration at a cut-off level of >29.8, with 91.9% sensitivity, 87.5% specificity, 88% PPV and 91.5% NPV (AUC=0.96, p-value<0.001) (Table 4).

**Table 4:** Diagnostic performance of serum CRP in discriminating patients with and without CT infiltration

	Cut off	AUC	Sensitivity	Specificity	PPV	NPV	p-value
CRP	> 29.8	0.96	91.9%	87.5%	88%	91.5%	< 0.001

## DISCUSSION

COVID-19 patients often require stratification based on the presence or absence of pneumonic infiltrates, along with severity grading for those with lung involvement. Such classification is crucial for tailoring treatment plans, especially for medications like anticoagulants and steroids, typically indicated for patients with pneumonia but not those without it. Such categorization requires an armamentarium of laboratory tests and thoracic imaging, not without hazards and additional costs.

In the face of the overwhelming COVID-19 pandemic and the challenges it presents to healthcare systems, there is an urgent demand for rapid, simple, and dependable measures to aid in the early detection of pneumonic infiltrates during the initial evaluation of patients. In this study, we explored the potential of CRP as a readily available and cost-effective laboratory marker for this purpose. CRP is a well-known acute phase reactant that can be easily measured, and its levels have been correlated with disease severity in COVID-19. In this prospective cross-sectional study, we enrolled a total of 210 patients who tested positive for COVID-19 through PCR analysis. The study group consisted of 136 males and 74 females, all non-smokers, and previously had no significant health issues. Their ages ranged from 18 to 60 years, with a mean age of 39.7±13.04 years. Among these patients, 48 exhibited normal CT chest findings, while the remaining 162 showed evidence of pneumonic infiltrates (Table 1).

The mean value of CRP levels was found to be higher in males (40.3±14.3) compared to females (36.6±15.2), although the difference was not statistically significant (Table 2). This observation aligns with a study by Lau ES *et al*. [5], which also reported that in patients with COVID-19, CRP levels were generally higher in men compared to women, even after adjusting for standard CRP measurements.

The mean value of CRP level was higher in patients with pneumonic infiltrates (45.02±10.2) compared to those without pneumonic infiltrates (18.8±7.8), with a highly statistically significant difference (Table 3). These results are consistent with the observations made by previous studies. For instance, L. Wang [6] reported a strong correlation between CRP levels and the extent of pulmonary infiltrates during the early phase of COVID-19 infection. Similarly, Wei Chen *et al*. [7] found a direct association between the extent of severity of CT infiltration and the rising level of CRP regardless of patient age and lymphocyte count. Moreover, Mayada Moneer *et al*. [8] concluded that serum CRP is a simple and effective laboratory marker for identifying patients at risk of developing a severe form of COVID-19. In a study by Gao Y *et al*. [9], an average CRP level of 39.4 mg/L was detected in patients with severe COVID-19 infection, while patients with mild infection had a CRP level of 18.8 mg/L, which closely aligns with the findings of our current study.

In this study, a CRP cut-off value of >29.8 mg/L was found to be indicative of the presence of pneumonic infiltrates with a sensitivity of 91.9% and specificity of 87.5% (Table 4). These findings are in line with previous research supporting the utility of CRP as a predictive marker for disease progression in COVID-19 patients. Wang G. *et al*. [10] suggested that a cut-off level of 26.9 mg/L for CRP may be used to anticipate the likelihood of disease progression in non-severe COVID-19 cases. Additionally, Bohdana Basina *et al*. [11] reported that elevated CRP concentrations above 53mg/L were associated with a greater tendency for disease progression. Furthermore, Mahmoud Sadeghi *et al*. [12] found that patients with serum CRP concentrations exceeding 64.75-mg/L had a higher risk of developing a severe form of COVID-19 disease.

Identifying a CRP cut-off value of >29.8 mg/L in our study has practical implications for the daily management of COVID-19 patients. By using this threshold, unnecessary CT chest examinations can be avoided in a significant number of patients, leading to reduced hazards, costs, and stress for both patients and healthcare providers. Unwanted CT chest scans may not only expose patients to additional radiation but also put a strain on the resources and capacity of the radiology department.

Limitations of this study may include the relatively small number of participants and the single ethnicity of patients, which may limit the generalizability of such results. We recommend conducting large, multicenter studies with diverse populations to validate and expand upon our findings.

## CONCLUSION

CRP is a relatively simple and inexpensive test that can suggest to a great extent if pneumonic infiltrates were present in patients with early COVID-19 infection who were still not hypoxemic. The level of >29.8 mg/L could be taken as a reference value, although further tests are needed to assess the reliability of this value.

## Data Availability

The datasets generated and/or analyzed during the current study are not publicly available but are available from the corresponding author upon reasonable request.
